# Risk of acute kidney injury and mortality in patients vaccinated against COVID-19

**DOI:** 10.7150/ijms.119217

**Published:** 2025-10-27

**Authors:** Po-Yu Tsai, Yu-Hsun Wang, Jing-Yang Huang, Shun-Fa Yang, Sheng-Wen Wu

**Affiliations:** 1 Institute of Medicine, Chung Shan Medical University, Taichung, Taiwan.; 2 Division of Nephrology, Department of Internal Medicine, Chung Shan Medical University Hospital, Taichung, Taiwan.; 3 Department of Medical Research, Chung Shan Medical University Hospital, Taichung, Taiwan.; 4 School of Medicine, Chung Shan Medical University, Taichung, Taiwan.

**Keywords:** COVID-19, vaccination, renal dysfunction, acute kidney injury, dialysis

## Abstract

Several types of vaccines have been developed to manage the coronavirus disease 2019 (COVID-19) pandemic. Although COVID-19 vaccines have demonstrated reasonable efficacy, cases of cardiac, vascular and renal complications have been observed. Herein, the association between COVID-19 vaccination and subsequent renal dysfunction and mortality was analyzed using data collected from TriNetX. A retrospective cohort study was conducted of patients vaccinated against COVID-19. After exclusion and matching, a total of 1,454,791 patients each were included in the vaccinated and unvaccinated groups. The primary outcome measured was renal dysfunction and mortality. In total, 15,809 and 11,801 of AKI, and 1,513 and 697 of dialysis treatment were observed in the vaccinated and unvaccinated groups, respectively. After one year, the vaccinated group exhibited significantly higher incidences of AKI (HR: 1.20, 95% CI:1.18-1.23), and dialysis (HR: 1.84, 95% CI:1.68-2.01) than the unvaccinated group. The vaccinated group exhibited significant lower incidences of mortality (HR: 0.88, 95% CI:0.85-0.91) than the unvaccinated group. The cumulative probability of AKI and dialysis was significantly higher in the vaccinated group than the unvaccinated group. In conclusion, COVID-19 vaccination was associated with a higher risk of developing acute kidney injury, but lower rate of mortality.

## Introduction

Severe acute respiratory syndrome coronavirus 2 (SARS-CoV-2) is a highly transmissible virus responsible for the outbreak of coronavirus disease 2019 (COVID-19) 1, 2. COVID-19 causes considerable damage to multiple organs, including the lungs, heart, kidneys, liver, and vasculature 3. The main mechanisms through which SARS-CoV-2 causes damage involve a combination of direct viral effects, immune responses, and systemic inflammation 4-6. The overall mortality rate of COVID-19 was reported to be approximately 1%-2% in most countries and up to 8% in developing countries 7.

Several vaccines have been developed for COVID-19 prevention since late 2020, including messenger RNA (mRNA) vaccines (BNT162b2 [Pfizer-BioNTech] and mRNA-1273 [Moderna]) and adenoviral vector vaccines (ChAdOx1-S [Oxford/AstraZeneca] and Ad26.COV2.S [Janssen/Johnson & Johnson]) 8, 9. Widespread inoculation with these vaccines has substantially reduced the incidence of COVID-19 10. Both mRNA and adenoviral vector COVID-19 vaccines have demonstrated more than 80% efficacy 11, 12. The BioNTech vaccine exhibited a slightly higher efficiency than other vaccines available 13.

Despite the high efficacy of COVID-19 vaccines reported in previous studies 11, adverse effects have also been documented 14, 15. Myocarditis has been reported following administration of mRNA-based COVID-19 vaccines 16, while immune thrombotic thrombocytopenia has been observed in individuals receiving adenoviral vector vaccines 14. In addition, cases of acute kidney injury (AKI) have been reported after COVID-19 vaccination 17. Whether COVID-19 vaccination is associated with renal dysfunction remains uncertain. Given the relatively small sample sizes of previous studies, investigation of this association in a higher number of patients is needed.

Consequently, we evaluated the association between COVID-19 vaccination and subsequent renal dysfunction, including AKI and dialysis. Correlations between other health parameters and renal dysfunction were also evaluated.

## Materials and Methods

### Data sources

In this retrospective cohort study, we collected data from the TriNetX analytics platform, an online database containing the deidentified electronic health records of more than 100 million patients from multiple regions. The TriNetX project was initiated by a collaborative network of 67 health care institutes, primarily large tertiary medical centers across the US with both outpatient and inpatient departments. The TriNetX database thus encompasses a diverse range of geographical regions, ethnic populations, age ranges, income levels, and insurance classes. The insurance classes in the TriNetX database comprise commercial, government-provided (Medicare and Medicaid), workers' compensation, military and veterans affairs, and self-paid insurance and uninsured patients. The information collected in the TriNetX database includes age and sex, length of hospitalization (if required), diagnoses (recorded with International Classification of Diseases, Tenth Revision, Clinical Modification (ICD-10-CM) codes), laboratory measurements, image codes, medical procedures (recorded with International Classification of Diseases, Ninth Revision, Procedure Coding System codes), and medication use (recorded with Anatomical Therapeutic Chemical codes). The study was approved by the institutional review board of Chung Shan Medical University Hospital (CS2-24180) and the National Health Insurance Administration of Taiwan. Both institutions waived the requirement for written informed consent.

### Study participants

**Figure 1:**
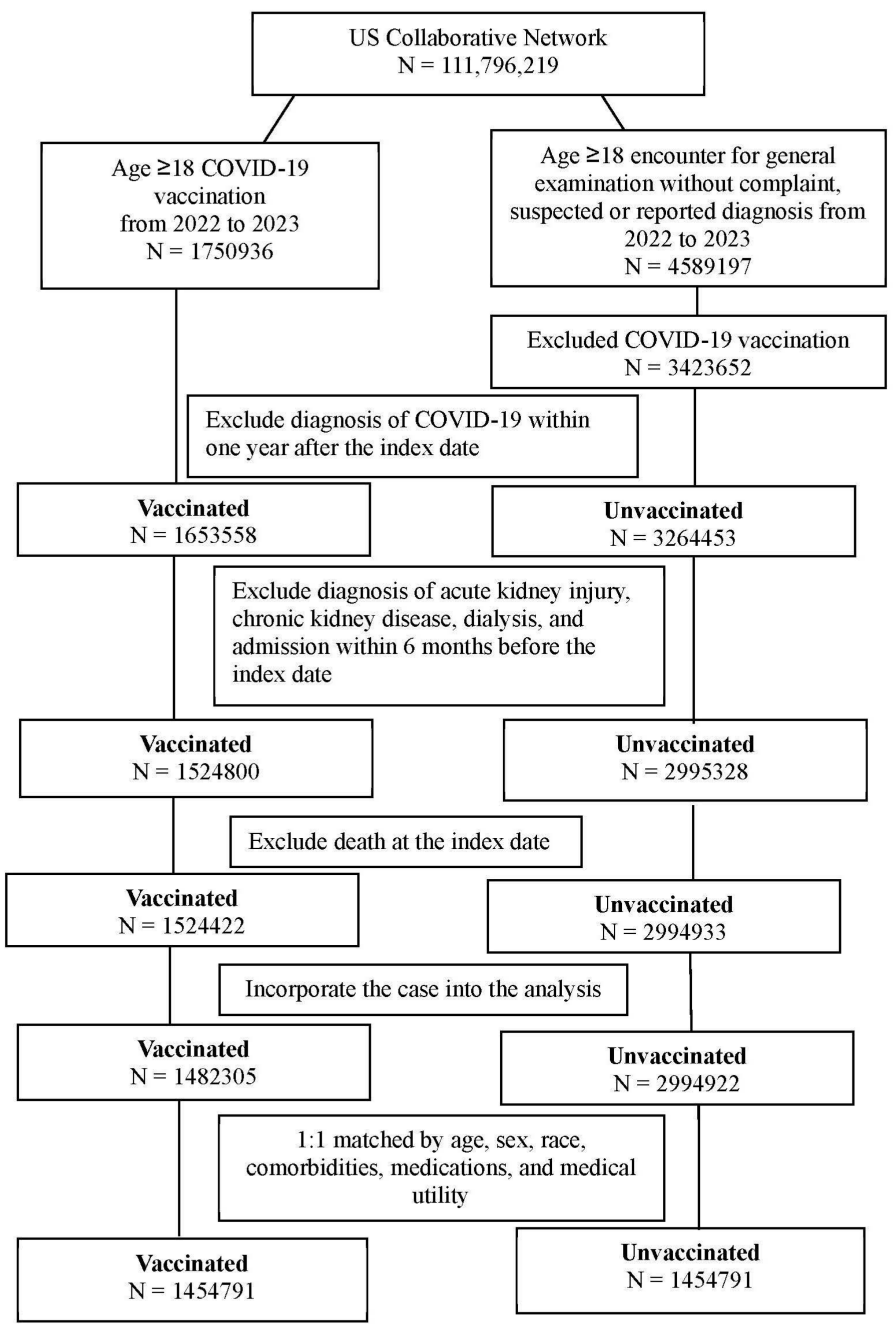
** The flowchart of participant selection.** COVID-19: coronavirus disease 2019, N: number

We conducted a retrospective cohort study of patients who (1) were aged ≥18 years old and (2) received a BioNTech, Moderna, or Janssen SARS-CoV-2 vaccine between 2022 and 2023. The index date was defined as the date of SARS-CoV-2 vaccination. To standardize the general health condition of the study population and establish the time sequence between COVID-19 vaccination and subsequent renal dysfunction, the following exclusion criteria were adopted: (1) mortality within three months after index date, (2) AKI diagnosis within 6 months before index date, (3) chronic kidney disease diagnosis within 6 months before index date, (4) dialysis treatment within 6 months before index date, (5) hospital admission within 6 months before index date, and (6) COVID-19 diagnosis within one year after index date. Additionally, a group of patients who met the following criteria were included for comparison: (1) aged ≥18 years, (2) had no reported concerns during general examinations and no suspected or reported diagnoses from 2022-2023, and (3) remained unvaccinated against COVID-19. The same exclusion criteria were applied to the unvaccinated group. Subsequently, propensity score matching (PSM) was performed to facilitate comparison between the vaccinated and unvaccinated groups. PSM between vaccinated and unvaccinated patients was conducted at a 1:1 ratio using the built-in function in the TriNetX database, which accounts for age, sex, race, comorbidities, medications, and medical utility. The PSM procedure applied a greedy nearest neighbor matching algorithm with a caliper of 0.1 pooled standard deviations. A final total of 1,454,791 patients were selected for each group. The participant selection flowchart is displayed in Figure 1.

### Primary outcome

The primary outcome examined in this study was renal dysfunction, encompassing the diagnosis of AKI and dialysis initiation. A patient was considered to be eligible and have AKI if they had a (1) diagnosis of AKI according to ICD-10-CM codes; (2) laboratory codes for complete blood cell count, white blood cell differentiate count, blood urea nitrogen, and creatinine tests before AKI diagnosis; and (3) a nephrologist or internal medicine physician as their diagnosis practitioner. A patient was considered to be eligible and undergoing dialysis treatment if they had a (1) diagnosis of AKI, chronic kidney disease, or other renal diseases according to ICD-10-CM codes; (2) laboratory codes for complete blood cell count, white blood cell differentiate count, blood urea nitrogen, and creatinine tests before AKI diagnosis; (3) procedure codes for dialysis; and (4) dialysis treatment arranged by a nephrologist or internal medicine physician. To better establish the time sequence between COVID-19 vaccination and renal dysfunction, only renal dysfunction episodes occurring after the index date were included in the study.

### Confounding factors

The following confounding factors were adjusted for: age, sex, race, and comorbidities including hypertension, dyslipidemia, type 2 diabetes mellitus (T2DM), obesity, ischemic heart disease, nicotine dependence, anemia, cerebrovascular disease, urolithiasis, and autoimmune diseases (Sjögren's syndrome, rheumatoid arthritis, systemic lupus erythematosus, and ankylosing spondylitis). Medication use was also considered, including lipid-modifying agents, NSAIDs, corticosteroids, antihypertensives (angiotensin agents, beta-blockers, calcium channel blockers), metformin, GLP-1 analogues, SGLT2 inhibitors, and both topical and systemic antibiotics. Exposure to nephrotoxic contrast agents was accounted for, including low-osmolar contrast media (300-399 mg/ml iodine), perflutren lipid microspheres, octafluoropropane microspheres, and Tc-99m from non-highly enriched uranium sources. Patients were followed until renal dysfunction, death, withdrawal from the health insurance program, or December 21, 2023, whichever occurred first.

### Statistical analysis

All statistical analyses were conducted within the TriNetX platform using SAS version 9.4 (SAS Institute, Cary, NC, USA). Descriptive analysis used to summarize the baseline characteristics of both groups. The balance of baseline characteristics between both groups was assessed using the standardized mean difference (SMD). A SMD more than 0.1 indicated a significant difference between groups. Cox proportional hazards regression was employed to compare the incidence of renal dysfunction between groups, producing hazard ratios (HR) and 95% confidence intervals (CI) after adjustment for confounding factors. After a Kaplan-Meier curve was constructed, a log-rank test was used to compare the cumulative incidences of renal dysfunction events. In the sensitivity analysis, patients in both groups were categorized into subgroups according to age, sex, race, and comorbidities. Cox proportional hazard regression was then applied to analyze the risk of renal dysfunction in each subgroup, the association between COVID-19 vaccination and renal dysfunction, and potential differences in this association based on the type of COVID-19 vaccine administered. Values of P < 0.05 were considered statistically significant, with a P value less than 0.001 displayed as P < 0.001.

## Results

Table 1 presents the baseline characteristics of both groups. The mean age at index (53.92 ± 18.33 and 53.76 ± 18.19 years in the vaccinated and unvaccinated groups, respectively) did not differ significantly between groups (SMD = 0.009). Likewise, between-group similarities were observed in race, medical utility, and sex distribution (all SMD < 0.1). All included comorbidities exhibited similar distributions between the vaccinated and unvaccinated groups (SMD < 0.1). Nonsignificant differences in the proportion of patients administered medical prescriptions, antibiotics, and contrast media were observed between the vaccinated and unvaccinated groups (all SMD < 0.1, Table 1).

**Figure 2:**
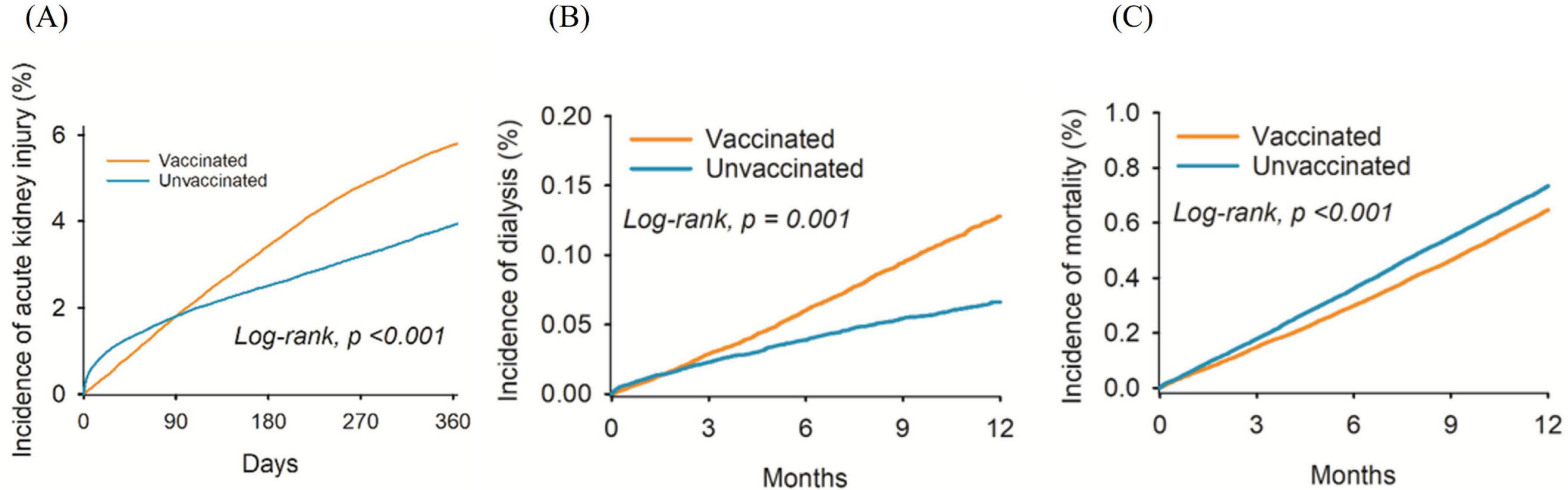
** The cumulative probability of (A) acute kidney injury, (B) dialysis, and (C) mortality between the two groups**.

Renal dysfunction diagnoses in the vaccinated and unvaccinated groups at different time periods are illustrated in Table 2. After a one-year follow-up period, we observed 15,809 and 11,081 cases of AKI, and 1,513 and 697 cases of dialysis treatment in the vaccinated and unvaccinated groups, respectively (Table 2). After adjustment for confounding factors, the incidence of AKI (HR: 1.20, 95% CI: 1.18-1.23), and dialysis (HR: 1.84, 95% CI: 1.68-2.01) was significantly higher in the vaccinated than in the unvaccinated group (Table 2). At the one-year follow-up, the number of deaths among vaccinated individuals was 7,693, while the number of deaths among unvaccinated individuals was 7,364. The incidence of mortality in vaccinated individuals was lower than unvaccinated individuals (HR: 0.88, 95% CI: 0.85-0.91). The cumulative incidence of AKI (Figure 2A) and dialysis (Figure 2B) were also significantly higher in the vaccinated group than the unvaccinated group. However, the probability of mortality was lower in in the vaccinated group than the unvaccinated group (P < 0.001) (Figure 2C).

In the subgroup analysis, the risk of AKI was significantly higher in vaccinated than in unvaccinated patients regardless of baseline characteristics, except in patients with systemic lupus erythematous or ankylosing spondylitis (Table 3). The incidence of dialysis was significantly higher in vaccinated than in unvaccinated patients regardless of baseline characteristics, except in patients with nicotine dependence, Sjögren syndrome, rheumatoid arthritis, systemic lupus erythematous, or ankylosing spondylitis (Table 4). Among different vaccines, the risk of developing AKI and requiring dialysis among BioNTech (HR: 1.51, 95% CI: 1.44-1.58) and Moderna vaccine (HR: 1.37, 95% CI: 1.28-1.47) recipients is significantly higher than that of unvaccinated individuals. The risk of death among BioNTech vaccine recipients is higher than that of unvaccinated individuals (HR: 1.20, 95% CI: 1.13-1.27). In contrast, the risk of death among Moderna vaccine recipients is lower than that of unvaccinated individuals (HR: 0.82, 95% CI: 0.75-0.90) (Table 5).

## Discussion

COVID-19 vaccination was associated with a higher risk of subsequent renal dysfunction, including AKI and dialysis treatment. The cumulative incidence of renal dysfunction was significantly higher in vaccinated than in unvaccinated patients.

Prior studies have indicated that COVID-19 vaccines can damage several tissues 18-20. The main pathophysiological mechanism of COVID-19 vaccine-related complications involve vascular disruption 21. COVID-19 vaccination can induce inflammation through interleukins and the nod-like receptor family pyrin domain-containing 3, an inflammatory biomarker 22. In another study, thrombosis episodes were observed in patients who received different COVID-19 vaccines 23. Additionally, mRNA COVID-19 vaccines have been associated with the development of myocarditis and related complications 19. The Pfizer-BioNTech and Moderna vaccines can also contribute to the development of myocarditis and pericarditis 16. Moreover, a correlation was observed between cerebrovascular diseases and prior mRNA COVID-19 vaccination 21, and coagulation defects were observed in patients who received adenoviral vector COVID-19 vaccines 14. Adenoviral vector COVID-19 vaccines have also been associated with a higher risk of Guillain-Barré syndrome 24. Neurological complications have been reported in patients vaccinated against COVID-19 25. The development of renal dysfunction can be affected by several biochemical factors 26. In turn, AKI can increase systemic inflammation 27 and impair the vasculature and red blood cell aggregation 28, 29. Given that the mechanism underlying COVID-19 vaccine-related complications corresponds to the pathophysiology of kidney disease 21, 22, 27, 28, we hypothesized that COVID-19 vaccination may cause renal dysfunction, which was supported by the results of this study.

In this study, COVID-19 vaccination was correlated with a higher incidence of renal dysfunction, including AKI and dialysis treatment. In a prior study, COVID-19 vaccination was associated with a higher risk of urological complication as voiding symptom and hematuria 30. Another study indicated that mRNA and adenoviral vector COVID-19 vaccines increased AKI risk in patients of Asian descent 17. Moreover, Lim et al. reported a case of acute interstitial nephritis with acute kidney injury in a young, healthy individual following administration of the COVID-19 vaccine 31.

However, a national population-based study from South Korea reported no evidence of an increased risk of adverse events following BNT162b2 vaccination 32. Nevertheless, the correlation between COVID-19 vaccination and renal dysfunction has not been investigated in detail. The results of this study preliminarily suggest a positive correlation between COVID-19 vaccination and subsequent renal dysfunction. We excluded renal dysfunction episodes that occurred before COVID-19 vaccination to better establish the time sequence between vaccination and renal dysfunction. Additionally, we adjusted for the following confounding factors in a Cox proportional hazard regression: hypertension, T2DM, cardiovascular diseases, overweight and obesity, and the administration of antibiotics or contrast media 33, 34. Consequently, COVID-19 vaccination was evaluated as an independent risk factor for subsequent renal dysfunction. The reduced risk of AKI within one month after vaccination may be attributed to lower mortality and fewer severe infections in vaccinated individuals, decreasing exposure to common triggers of kidney injury, such as sepsis and systemic inflammation. Three months after vaccination, the incidence of AKI and dialysis treatment were significantly higher in the vaccinated group than the unvaccinated group. Moreover, the cumulative incidences of AKI and dialysis treatment were significantly higher in the vaccinated group than the unvaccinated group. These results suggest that the influence of COVID-19 vaccination on renal dysfunction risk is persistent and may increase with time. The reason for this finding requires further validation.

In the subgroup analysis, a significantly higher risk of renal dysfunction, including AKI and dialysis, was observed for most subgroups of vaccinated patients compared with subgroups of unvaccinated patients, which few studies have previously investigated. One study indicated hypertension was a significant risk factor for chronic kidney disease 33. Additionally, T2DM is associated with higher incidence of kidney disease 26, and advanced-stage diabetic kidney disease may require dialysis management 35. Ischemic heart disease may be correlated with a higher risk of mortality in patients with renal disease 33. AKI has also commonly been observed in patients with autoimmune diseases 36. Accordingly, renal dysfunction may be reasonably assumed to appear more frequently in vaccinated patients with known risk factors for kidney disease compared with unvaccinated patients. Notably, several subgroups of the vaccinated population in our study exhibited a similar risk of requiring dialysis treatment to subgroups of the unvaccinated population, whereas AKI risk was significantly higher vaccinated than unvaccinated subgroups. These conflicting findings may be explained by the lower number of patients receiving dialysis than those diagnosed with AKI. In several subgroups, the number of patients receiving dialysis was less than 10, potentially creating statistical bias. A similar bias may have influenced the sensitivity analysis for vaccine types, in which the lower renal outcome numbers in patients who received the Janssen COVID-19 vaccine exhibited nonsignificant correlation with AKI development. The similar relationship to renal dysfunction observed among the three COVID-19 vaccines implies that the method of vaccine production did not affect the risk of subsequent renal dysfunction.

Epidemiological evidence suggests the COVID-19 pandemic is the largest pandemic in recent decades 2. By 2020, the COVID-19 pandemic had affected approximately 5.85 million people worldwide and caused an estimated 359,000 deaths 1. Even as the pandemic subsided 8, vaccination was recommended to prevent infection with COVID-19 variants 9, 37. Kidney disease is one of the most common noncommunicable diseases in the world 38. The prevalence of kidney disease is approximately 13.6% in the American population 39 and approximately 400 cases per million in individuals of Asian or Mexican descent 26. For severe renal injury, dialysis is typically recommended if renal transplant is not performed 40, 41 Dialysis can create tremendous medical costs 42, 43. Given that both COVID-19 infection and renal dysfunction affect many people and can cause severe complications, including mortality, the relationship between COVID-19 vaccination and renal dysfunction merits investigation. Nevertheless, COVID-19 vaccination has been shown to significantly reduce the risk of mortality, primarily by preventing severe SARS-CoV-2 infection and its associated complications (Table 2).

This study has several limitations. First, as a claims database, the TriNetX platform only provides diagnosis, examination, procedure, and medication codes, although laboratory examination results are also available. Consequently, certain crucial information could not be investigated, such as the COVID-19 vaccination dose, antibody titer after COVID-19 vaccination, development process and clinical symptoms of renal dysfunction, medical compliance and treatment response for renal dysfunction, dialysis procedure details and frequency, detailed comorbidity data, and medication use. Second, although we conducted PSM, the retrospective nature of our study may have reduced participant homogeneity. Additionally, some over-the-counter drugs for pain control that may damage the kidney were not included in the TriNetX database. Finally, because the exact molecular pathway through which COVID-19 vaccination induces renal dysfunction could not be assessed through database research, the integrity of the relationship we observed between COVID-19 vaccination and renal dysfunction requires further validation.

In this study, COVID-19 vaccination plays a critical role in reducing mortality. However, COVID-19 vaccination was correlated with the subsequent development of renal dysfunction after adjustment for multiple confounders. Furthermore, this relationship became more prominent with time following vaccination and was not affected by the type of COVID-19 vaccine administered. Consequently, periodic renal examination may be advisable for patients who received a COVID-19 vaccine. Further large-scale prospective studies are required to clarify the effect of COVID-19 vaccination on renal dysfunction.

## Figures and Tables

**Table 1:** Demographic characteristics of vaccinated and unvaccinated groups.

Characteristics	Vaccinated N = 1454791	Unvaccinated N = 1454791	SMD
**Age at Index**	53.92 ± 18.33	53.76 ± 18.19	0.009
**Sex**			
Female	848042 (58.29)	855832 (58.83)	0.011
Male	589392 (40.51)	581611 (39.98)	0.011
**Race**			
White	906117 (62.29)	888756 (61.09)	0.025
African American	203666 (14.00)	212616 (14.62)	0.018
Asian	99214 (6.82)	104727 (7.20)	0.015
**Comorbidities**			
Hypertensive diseases	307490 (21.14)	317370 (21.82)	0.017
Dyslipidemia	289250 (19.88)	294978 (20.28)	0.010
T2DM	123191 (8.47)	128857 (8.86)	0.014
Overweight and obesity	117894 (8.10)	121318 (8.34)	0.009
Ischemic heart diseases	61691 (4.24)	64170 (4.41)	0.008
Nicotine dependence	43694 (3.00)	42755 (2.94)	0.004
Anemias	32908 (2.26)	32008 (2.20)	0.004
Cerebrovascular diseases	26956 (1.85)	27012 (1.86)	0.000
Urolithiasis	17018 (1.17)	15608 (1.07)	0.009
Sjögren syndrome	5350 (0.37)	4622 (0.32)	0.009
Rheumatoid arthritis	10568 (0.73)	9604 (0.66)	0.008
Systemic lupus erythematosus	3335 (0.23)	3044 (0.21)	0.004
Ankylosing spondylitis	1274 (0.09)	1008 (0.07)	0.007
**Medication**			
Lipid modifying agents	223277 (15.35)	200867 (13.81)	0.044
NSAID	182806 (12.57)	183265 (12.60)	0.001
Corticosteroids	168434 (11.58)	170979 (11.75)	0.005
Angiotensin agents	187208 (12.87)	194587 (13.38)	0.015
Beta blocking agents	131311 (9.03)	117812 (8.10)	0.033
Calcium channel blockers	107907 (7.42)	100579 (6.91)	0.020
Metformin	77167 (5.30)	68744 (4.73)	0.027
GLP-1 analogues	29653 (2.04)	26846 (1.85)	0.014
SGLT2 inhibitors	19180 (1.32)	16922 (1.16)	0.014
**Medical utility**			
Ambulatory	989401 (68.01)	1004020 (69.02)	0.022
Emergency	136893 (9.41)	135476 (9.31)	0.003
Inpatient Encounter	27489 (1.89)	25763 (1.77)	0.009
**Antibiotics**			
Systemic antibiotic	334009 (22.96)	337218 (23.18)	0.005
Topical antibiotic	81821 (5.62)	80587 (5.54)	0.004
**Contrast media**	39172 (2.69)	46111 (3.17)	0.028
Low osmolar contrast material	39172 (2.69)	46111 (3.17)	0.028
Perflutren lipid	5322 (0.37)	3949 (0.27)	0.017
Octafluoropropane	1062 (0.07)	1169 (0.08)	0.003
Tc-99m	1087 (0.08)	5420 (0.37)	0.063

GLP-1: glucagon-like peptide-1, N: number, NSAID: non-steroidal anti-inflammatory drugs, SGLT2: sodium-glucose cotransporter 2, SMD: standardized mean difference, T2DM: type 2 diabetes mellitus.

**Table 2:** Risk of renal dysfunction including AKI and dialysis treatment, and mortality in different follow-up duration.

	Vaccinated		Unvaccinated		
	N	No. of event	N	No. of event	HR (95% C.I.)
**Acute kidney injury**					
1 month	1365390	1314	1365390	1526	0.85 (0.79-0.92)
3 months	1454791	3926	1454791	3774	0.99 (0.95-1.04)
6 months	1454791	7851	1454791	6555	1.10 (1.07-1.14)
9 months	1365390	11341	1365390	8723	1.16 (1.13-1.19)
12 months	1454791	15809	1454791	11081	1.20 (1.18-1.23)
**Dialysis**					
1 month	1365390	116	1365390	135	0.85 (0.66-1.09)
3 months	1454791	370	1454791	284	1.24 (1.06-1.45)
6 months	1454791	751	1454791	454	1.52 (1.35-1.71)
9 months	1365390	1154	1365390	593	1.73 (1.57-1.91)
12 months	1454791	1513	1454791	697	1.84 (1.68-2.01)
**Mortality**					
1 month	1365390	695	1365390	747	0.92 (0.83-1.02)
3 months	1454791	1910	1454791	2186	0.83 (0.78-0.88)
6 months	1454791	3739	1454791	4150	0.83 (0.79-0.86)
9 months	1365390	5701	1365390	5608	0.90 (0.87-0.94)
12 months	1454791	7693	1454791	7364	0.88 (0.85-0.91)

HR: hazard ratio, AKI: acute kidney injury, C.I.: confidence interval, N: number.

**Table 3:** Stratification analysis of risk of acute kidney injury among different group.

	Vaccinated		Unvaccinated		
	N	No. of event	N	No. of event	HR (95% C.I.)
Age					
18-64	986853	5848	986853	3632	1.35 (1.30-1.41)
≥65	457618	9344	457618	7289	1.13 (1.09-1.16)
Sex					
Female	873094	7263	873094	5063	1.22 (1.17-1.26)
Male	598558	8489	598558	5654	1.27 (1.23-1.31)
Race					
White	895661	9990	895661	6820	1.27 (1.24-1.31)
African American	203088	2995	203088	1827	1.32 (1.25-1.40)
Asian	99130	519	99130	272	1.63 (1.41-1.89)
Hypertensive diseases	292009	7070	292009	5260	1.17 (1.13-1.21)
Dyslipidemia	274773	5301	274773	3835	1.19 (1.14-1.24)
Type 2 diabetes mellitus	116871	3574	116871	2607	1.19 (1.13-1.25)
Overweight and obesity	111247	2089	111247	1551	1.16 (1.08-1.23)
Ischemic heart diseases	57984	2264	57984	1722	1.14 (1.07-1.22)
Nicotine dependence	39238	1096	39238	862	1.11 (1.02-1.21)
Sjögren syndrome	5164	98	5164	57	1.58 (1.14-2.18)
Rheumatoid arthritis	12834	297	12834	199	1.29 (1.08-1.55)
Systemic lupus erythematosus	3207	75	3207	62	1.06 (0.75-1.48)
Ankylosing spondylitis	1160	21	1160	22	0.80 (0.44-1.45)

HR: adjusted hazard ratio, C.I.: confidence interval, N: number.

**Table 4:** Stratification analysis of risk of dialysis among different group.

	Vaccinated		Unvaccinated		
	N	No. of event	N	No. of event	HR (95% C.I.)
Age					
18-64	986853	809	986853	324	2.10 (1.85-2.39)
≥65	457618	650	457618	343	1.67 (1.47-1.90)
Sex					
Female	873094	630	873094	309	1.74 (1.52-1.99)
Male	598558	863	598558	385	1.89 (1.68-2.13)
Race					
White	895661	776	895661	372	1.81 (1.60-2.05)
African American	203088	373	203088	207	1.48 (1.25-1.76)
Asian	99130	87	99130	22	3.38 (2.12-5.40)
Hypertensive diseases	292009	373	292009	225	1.45 (1.23-1.71)
Dyslipidemia	274773	256	274773	142	1.56 (1.27-1.91)
Type 2 diabetes mellitus	116871	267	116871	153	1.51 (1.24-1.84)
Overweight and obesity	111247	98	111247	57	1.47 (1.06-2.04)
Ischemic heart diseases	57984	134	57984	86	1.37 (1.04-1.79)
Nicotine dependence	39238	40	39238	30	1.16 (0.72-1.87)
Sjögren syndrome	5164	10	5164	10	3.21 (0.67-15.44)
Rheumatoid arthritis	12834	14	12834	10	1.22 (0.54-2.74)
Systemic lupus erythematosus	3207	12	3207	10	1.31 (0.53-3.21)
Ankylosing spondylitis	1160	10	1160	10	1.85 (0.17-20.37)

HR: hazard ratio, C.I.: confidence interval, N: number

**Table 5:** Analysis of risk of renal dysfunction including AKI and dialysis treatment among different vaccines.

Outcome	Vaccinated		Unvaccinated		HR (95% CI)
	N	Event	N	Event	
**AKI**					
BioNTech	384979	4839	384979	2825	1.51 (1.44-1.58)
Moderna	166402	2257	166402	1377	1.37 (1.28-1.47)
Janssen	3778	39	3778	18	1.73 (0.99-3.04)
**Dialysis**					
BioNTech	384979	415	384979	178	2.07 (1.74-2.47)
Moderna	166402	309	166402	82	3.15 (2.47-4.02)
Janssen^#^	3778	10	3778	10	4.50 (0.54-37.43)
**Mortality**					
BioNTech	384979	2553	384979	1860	1.20 (1.13-1.27)
Moderna	166402	903	166402	908	0.82 (0.75-0.90)
Janssen	3778	13	3778	15	0.69 (0.33-1.44)

HR: hazard ratio, AKI: acute kidney injury, CI: confidence interval, N: number. # If the patient's count is 1-10, the results indicate a count of 10.

## References

[1] Chu DK, Akl EA, Duda S, Solo K, Yaacoub S, Schünemann HJ (2020). Physical distancing, face masks, and eye protection to prevent person-to-person transmission of SARS-CoV-2 and COVID-19: a systematic review and meta-analysis. Lancet.

[2] Peeling RW, Heymann DL, Teo YY, Garcia PJ (2022). Diagnostics for COVID-19: moving from pandemic response to control. Lancet.

[3] Sushma DS, Jaiswal V, Kumar A, Asha S, Pal T (2022). Insights into Novel Coronavirus Disease 2019 (COVID-19): Current Understanding, Research, and Therapeutic Updates. Recent Pat Biotechnol.

[4] Ashraf A, Liaquat A, Shabbir S, Bokhari SA, Tariq Z, Furrukh Z (2023). High level of lactate dehydrogenase and ischaemia-reperfusion injury regulate the multiple organ dysfunction in patients with COVID-19. Postgrad Med J.

[5] Topper MJ, Guarnieri JW, Haltom JA, Chadburn A, Cope H, Frere J (2024). Lethal COVID-19 associates with RAAS-induced inflammation for multiple organ damage including mediastinal lymph nodes. Proc Natl Acad Sci U S A.

[6] Balan C, Ciuhodaru T, Bubenek-Turconi SI (2023). Kidney Injury in Critically Ill Patients with COVID-19 - From Pathophysiological Mechanisms to a Personalized Therapeutic Model. J Crit Care Med (Targu Mures).

[7] Ochani R, Asad A, Yasmin F, Shaikh S, Khalid H, Batra S (2021). COVID-19 pandemic: from origins to outcomes. A comprehensive review of viral pathogenesis, clinical manifestations, diagnostic evaluation, and management. Infez Med.

[8] Feikin DR, Higdon MM, Abu-Raddad LJ, Andrews N, Araos R, Goldberg Y (2022). Duration of effectiveness of vaccines against SARS-CoV-2 infection and COVID-19 disease: results of a systematic review and meta-regression. Lancet.

[9] Krause PR, Fleming TR, Longini IM, Peto R, Briand S, Heymann DL (2021). SARS-CoV-2 Variants and Vaccines. N Engl J Med.

[10] Hadj Hassine I (2022). Covid-19 vaccines and variants of concern: A review. Rev Med Virol.

[11] Fiolet T, Kherabi Y, MacDonald CJ, Ghosn J, Peiffer-Smadja N (2022). Comparing COVID-19 vaccines for their characteristics, efficacy and effectiveness against SARS-CoV-2 and variants of concern: a narrative review. Clin Microbiol Infect.

[12] Beladiya J, Kumar A, Vasava Y, Parmar K, Patel D, Patel S (2024). Safety and efficacy of COVID-19 vaccines: A systematic review and meta-analysis of controlled and randomized clinical trials. Rev Med Virol.

[13] Abdel-Moneim AS, Abdelwhab EM, Memish ZA (2021). Insights into SARS-CoV-2 evolution, potential antivirals, and vaccines. Virology.

[14] Tregoning JS, Flight KE, Higham SL, Wang Z, Pierce BF (2021). Progress of the COVID-19 vaccine effort: viruses, vaccines and variants versus efficacy, effectiveness and escape. Nat Rev Immunol.

[15] Mushtaq HA, Khedr A, Koritala T, Bartlett BN, Jain NK, Khan SA (2022). A review of adverse effects of COVID-19 vaccines. Infez Med.

[16] Bozkurt B, Kamat I, Hotez PJ (2021). Myocarditis With COVID-19 mRNA Vaccines. Circulation.

[17] Chen CC, Yang SS, Hsu YJ, Sung CC, Chu P, Wu CC (2023). Acute kidney disease following COVID-19 vaccination: a single-center retrospective study. Front Med (Lausanne).

[18] Sharif N, Alzahrani KJ, Ahmed SN, Dey SK (2021). Efficacy, Immunogenicity and Safety of COVID-19 Vaccines: A Systematic Review and Meta-Analysis. Front Immunol.

[19] Barouch DH (2022). Covid-19 Vaccines - Immunity, Variants, Boosters. N Engl J Med.

[20] Zhu Y, Ouyang X, Zhang D, Wang X, Wu L, Yu S (2024). Alopecia areata following COVID-19 vaccine: a systematic review. Eur J Med Res.

[21] Yasmin F, Najeeb H, Naeem U, Moeed A, Atif AR, Asghar MS (2023). Adverse events following COVID-19 mRNA vaccines: A systematic review of cardiovascular complication, thrombosis, and thrombocytopenia. Immun Inflamm Dis.

[22] Chen Y, Xu Z, Wang P, Li XM, Shuai ZW, Ye DQ (2022). New-onset autoimmune phenomena post-COVID-19 vaccination. Immunology.

[23] Faksova K, Walsh D, Jiang Y, Griffin J, Phillips A, Gentile A (2024). COVID-19 vaccines and adverse events of special interest: A multinational Global Vaccine Data Network (GVDN) cohort study of 99 million vaccinated individuals. Vaccine.

[24] Keh RYS, Scanlon S, Datta-Nemdharry P, Donegan K, Cavanagh S, Foster M (2023). COVID-19 vaccination and Guillain-Barré syndrome: analyses using the National Immunoglobulin Database. Brain.

[25] Allahyari F, Molaee H, Hosseini Nejad J (2023). Covid-19 vaccines and neurological complications: a systematic review. Z Naturforsch C J Biosci.

[26] Levey AS, Coresh J (2012). Chronic kidney disease. Lancet.

[27] Bellomo R, Kellum JA, Ronco C (2012). Acute kidney injury. Lancet.

[28] Koza Y (2016). Acute kidney injury: current concepts and new insights. J Inj Violence Res.

[29] Varrier M, Ostermann M (2014). Novel risk factors for acute kidney injury. Curr Opin Nephrol Hypertens.

[30] Shim SR, Kim KT, Park E, Pyun JH, Kim JH, Chung BI (2023). Urological complications after COVID 19 vaccine according to age, sex and manufacturer. World J Urol.

[31] Lim J, Paek JH, Shin HC, Park WY, Jin K, Choe M (2024). Acute interstitial nephritis with acute kidney injury after COVID-19 vaccination: a case report. Clin Exp Vaccine Res.

[32] Choe YJ, Ahn YH, Gwak E, Jo E, Kim J, Choe SA (2024). Safety of BNT162b2 mRNA COVID-19 vaccine in children with chronic kidney disease: a national population study from South Korea. Pediatr Nephrol.

[33] Kalantar-Zadeh K, Jafar TH, Nitsch D, Neuen BL, Perkovic V (2021). Chronic kidney disease. Lancet.

[34] Levey AS, James MT (2017). Acute Kidney Injury. Ann Intern Med.

[35] Umanath K, Lewis JB (2018). Update on Diabetic Nephropathy: Core Curriculum 2018. Am J Kidney Dis.

[36] Márquez-Macedo SE, Perez-Arias AA, Pena-Vizcarra Ó R, Zavala-Miranda MF, Juárez-Cuevas B, Navarro-Gerrard MA (2023). Predictors of treatment outcomes in lupus nephritis with severe acute kidney injury and requirement of dialytic support. Clin Rheumatol.

[37] Sachs JD, Karim SSA, Aknin L, Allen J, Brosbøl K, Colombo F (2022). The Lancet Commission on lessons for the future from the COVID-19 pandemic. Lancet.

[38] Couser WG, Remuzzi G, Mendis S, Tonelli M (2011). The contribution of chronic kidney disease to the global burden of major noncommunicable diseases. Kidney Int.

[39] Saran R, Li Y, Robinson B, Ayanian J, Balkrishnan R, Bragg-Gresham J (2015). US Renal Data System 2014 Annual Data Report: Epidemiology of Kidney Disease in the United States. Am J Kidney Dis.

[40] Elliott DA (2000). Hemodialysis. Clin Tech Small Anim Pract.

[41] KDOQI Clinical Practice Guideline for Hemodialysis Adequacy (2015). 2015 update. Am J Kidney Dis.

[42] Mehrotra R, Devuyst O, Davies SJ, Johnson DW (2016). The Current State of Peritoneal Dialysis. J Am Soc Nephrol.

[43] Himmelfarb J, Ikizler TA (2010). Hemodialysis. N Engl J Med.

